# Supplementary material for: Mouse liver assembloids model periportal architecture and biliary fibrosis
Source: Nature. 2025 May 29;644(8076):473–82. doi: 10.1038/s41586-025-09183-9 (PMC12350178; doi:10.1038/s41586-025-09183-9)
Supplement: Supplementary file 1 — Supplementary Methods, Supplementary Fig. 2, Supplementary Table 7 and references. [file 41586_2025_9183_MOESM1_ESM.docx]

**Supplementary Information**

**Mouse liver assembloids model periportal architecture and biliary fibrosis**

Anna M. Dowbaj, Aleksandra Sljukic, Armin Niksic, Cedric Landerer, Julien Delpierre, Haochen Yang, Aparajita Lahree, Ariane C. Kühn, David Beers, Helen M. Byrne, Sarah Seifert, Heather A. Harrington, Marino Zerial, Meritxell Huch

**Supplementary Figures - …………………………..…………………………………Page 2**

**Supplementary Methods ……...……………………………………………………….Page 2**

**Supplementary Tables Legends……...……………………………………………..…Page 2**

**Supplementary Videos Legends……………………………………………………….Page 3**

**Supplementary Figure 1 - Cytokine array raw data**

Cytokine array blots from 2 biological replicates, presented as raw scans of the signal, and overlay with the blot brightfield image. Images are annotated for the sample name. Supernatants from monocultures of CholOrg, or HepOrg or Msc cells or from assembloids from homeostatic or fibrotic-like conditions were analysed.

**Supplementary Figure 2 - Analysis of Morphological Differences Between Two Phenotypic Classes of Organoids.** (a) Schematic illustration of the DETECT (Detecting Temporal Shape Changes with the Euler Characteristic Transform) pipeline. The algorithm’s input takes segment- ed boundaries of organoids and computes the ECT (Euler Characteristic Transform) as collection of curves. These curves are then used to calculate the DETECT curve. The DETECT curves can be used as feature vectors for downstream visualisation algorithms and clustering algorithms, or to compute the pairwise DETECT metric heatmap. (b) Visualization of morphological variations using t-SNE based on DETECT calculations, with points coloured according to the shape type of the corresponding organoid. (c) Results of K-means clustering applied to DETECT calculations post-PCA reduction to two principal components. Each dot represents an organoid, labelled 'B' for ball-shaped and 'BG' for bubbly/grape-shaped. Crosses indicate the centroids of the respective clusters, with colours denoting the cluster assignment. (d) Heatmap of the DETECT metric distance for the DETECT results of each pair of organoids.

**Supplementary Methods**

Methods used in the manuscript referring to the Fiji stack files for Extended Data Figure 2a (Appendix A) and the description of the DETECT (Detecting Temporal Shape Changes with the Euler Characteristic Transform) method including the mathematical description (Appendix B and Appendix C). Supplementary Figure 2 and Supplementary Table 7 are provided as part of this section.

**Supplementary Tables Legends**

**Supplementary Table 1 - List of reagents used in this study**

- Supplementary Table 1-1. List of antibodies and dyes
- Supplementary Table 1-2. List of RT-qPCR primers
- Supplementary Table 1-3. List of commercial kits
- Supplementary Table 1-4. List of RNAscope reagents
- Supplementary Table 1-5. List of siRNA sequences
- Supplementary Table 1-6. List of cytokines
- Supplementary Table 1-7. List of blocking antibodies

**Supplementary Table 2 – bulk RNA sequencing TPM values**

TPM values from bulk RNA sequencing for each gene were used for heatmaps in Extended Data Figure 3b (HM-Wnt HepOrg *vs* freshly isolated hepatocytes); Extended Data Figure 5a (HM-Wnt and MM media HepOrg *vs* freshly isolated hepatocytes); Extended Data Figure 10a (7 days or 2.5 weeks homeostasis and fibrotic-like assembloids).

**Supplementary Table 3 – GSEA comparing cells from homeostasis-like assembloids**

Gene set enrichment analysis (GSEA) of hepatocytes, cholangiocytes and portal mesenchyme; Related to Extended Data Figure 7k-m. Databases used are GO_Biological_Process_2023 (GO_BP), GO_Cellular_Component_2023 (GO_CC) and GO_Molecular_Function_2023 (GO_MF).

**Supplementary Table 4 – LIANA Inferred cell-cell interactions**

4_1: Interactions from fibrosis-like assembloids and the bile duct ligation (BDL) and CCl4 models from Yang et al., 2021; Related to Figure 4d, Top 100 significant interactions from all models;

4_2: Interactions from fibrosis-like and homeostasis assembloids; Related to Extended Data Figure 12a; Top 30 significant interactions from all models.

**Supplementary Table 5 – GSEA comparing fibrosis-like *versus* homeostasis assembloids**

Related to Figure 4e; GSEA Homeostasis and Fibrosis-like Assembloids using KEGG, reactome and MSigDB_Hallmarks databases; Related to Extended Data Figure 10e-g; GSEA of cells from fibrosis-like versus homeostasis assembloids for hepatocytes (e), cholangiocytes (f), and mesenchyme (g).

**Supplementary Table 6 - Differential gene expression (DGE) between HepOrg and freshly isolated hepatocytes.**

Differential gene expression for comparison of HepOrg in HM-Wnt or MM media cultured at passage 2 for 1 week or 2 weeks, compared to freshly isolated hepatocytes.

**Supplementary Table 7 – K-mean clustering results of organoid shape analysis.**

Silhouette scores for the K-mean clustering results for each analysed organoid; B denotes ball-shaped hepatocyte organoids, R denotes bubbly-shaped organoids.

**Supplementary Videos Legends**

**Supplementary Video 1: Bubbly/grape-like shaped HepOrg metabolise 5-CFDA.**

Maximum intensity projection of a live cell imaging of a bubbly-shaped HepOrg at passage 2. HepOrg was treated with 5-CFDA (pseudo-coloured Royal LUT). Scale bar, 50 µm.

**Supplementary Video 2: Ball-shaped HepOrg cannot metabolise 5-CFDA**

Maximum intensity projection of a live cell imaging of a ball-shaped HepOrg at passage 2. HepOrg was treated with 5-CFDA (pseudo-coloured Royal LUT). Scale bar, 50 µm.

**Supplementary Video 3: HepOrg transport CLF.**

Maximum intensity projection of a live cell imaging of a bubbly-shaped HepOrg in MM media at passage 2, showing functional update and transport of CLF. Dyes are visualised with pseudo-coloured Royal LUT). Scale bar, 50 µm.

**Supplementary Video 4: HepOrg transport CMFDA.**

Maximum intensity projection of a live cell imaging of a bubbly-shaped HepOrg in MM media at passage 2, showing functional update and transport of CMFDA. Dyes are visualised with pseudo-coloured Royal LUT). Scale bar, 50 µm.

**Supplementary Video 5: HepOrg transport fluorescent Phosphatidylcholine (PC).**

Maximum intensity projection of a live cell imaging of a bubbly-shaped HepOrg in MM media at passage 2, showing functional update and transport of fluorescent PC. Dyes are visualised with pseudo-coloured Royal LUT). Scale bar, 50 µm.

**Supplementary Video 6: Periportal assembloid formation.**

Maximum intensity projection of assembloid formation 2 days after seeding. Time in hours. Nuclei are depicted in white (SPY620), mesenchyme cells in green (Pdgfra-H2BGF), cholangiocytes in magenta (mem-tdTomato). Brightfield is also shown. Assembloids can still recruit cells after being formed. Scale bar, 100 µm. Related to Extended Data Figure 7d.

**Supplementary Video 7: Periportal assembloid formation.**

Maximum intensity projection of assembloid formation 2 days after seeding. Time in hours. Nuclei are depicted in white (SPY620), mesenchyme cells in green (Pdgfra-H2BGF), cholangiocytes in magenta (mem-tdTomato). Brightfield is also shown. Scale bar, 100 µm. Related to Extended Data Figure 7d.

**Supplementary Video 8: Representative 3D-reconstruction showing the connection between bile canaliculi-bile duct in liver tissue.**

Animation of a 3D reconstruction of the interface of bile canaliculi-bile duct in mouse liver tissue. Example 1. Reconstruction was generated from multiphoton image showing a continuous lumen of bile canaliculi (CD13, green) entering bile duct (PCK, magenta). Hepatocytes, which connect their bile canaliculi to the bile duct lumen are visualised in different colours (red, yellow, cyan). Related to Fig. 3j.

**Supplementary Video 9: Representative 3D-reconstruction showing the connection between bile canaliculi-bile duct in a periportal liver assembloid.**

Animation of a 3D reconstruction showing interface of bile canaliculi-bile duct in a mouse periportal assembloid. Example 1. Reconstruction was generated from high-resolution Airyscan 3D image showing a continuous lumen of bile canaliculi (ZO-1, green) entering bile duct (KRT19, magenta). Hepatocytes, which connect their bile canaliculi to the bile duct lumen are visualised in different colours (red, yellow). Related to Fig. 3j.

**Supplementary Video 10: Representative 3D-reconstruction showing the connection between bile canaliculi-bile duct in liver tissue.**

Animation of a 3D reconstruction of the interface of bile canaliculi-bile duct in mouse liver tissue. Example 2. Reconstruction was generated from multiphoton image showing a continuous lumen of bile canaliculi (CD13, green) entering bile duct (PCK, magenta). Hepatocytes, which connect their bile canaliculi to the bile duct lumen are visualised in different colours (red, yellow, cyan). Related to Fig. 9b.

**Supplementary Video 11: Representative 3D-reconstruction showing the connection between bile canaliculi-bile duct in a periportal liver assembloid**

Animation of a 3D reconstruction showing interface of bile canaliculi-bile duct in a mouse periportal assembloid. Example 2. Reconstruction was generated from confocal image showing a continuous lumen of bile canaliculi (CD13, green) entering bile duct (nuc-tdTomato, magenta). Hepatocytes, which connect their bile canaliculi to the bile duct lumen are visualised in different colours (red, yellow, cyan). Related to Fig. 9b.

**Supplementary Video 12: Periportal assembloids transport bile acid analogue from bile canaliculi to bile duct lumens**.

Live imaging of the uptake and flow of the bile acid analogue cholyl-L-lysyl-fluorescein (CLF, pseudo-colour range) in assembloids shows functional transport of bile salts from bile canaliculi into the lumen of the bile duct lined by cholangiocytes (magenta). Time in minutes. Scale bar, 100 µm. Related to Fig. 3l.

**Supplementary Video 13: Periportal assembloids transport bile acid analogue from bile canaliculi to bile duct lumens**.

Live imaging of the uptake and flow of the bile acid analogue cholyl-L-lysyl-fluorescein (CLF, pseudo-colour range) in assembloids shows functional transport of bile salts from bile canaliculi into the lumen of the bile duct lined by cholangiocytes (magenta). Time in minutes. Scale bar, 100 µm. Related to Extended Data Fig. 9h.

**Supplementary Video 14: Structures with aberrant cholangiocyte ratio and non-physiological BC-BD connection do not transport bile acid analogue.**

Live imaging of the uptake and flow of the bile acid analogue cholyl-L-lysyl-fluorescein (CLF, pseudo-colour range). CLF uptake is not observed in structures with aberrant architecture where cholangiocytes are not embedded in the organoid (mem-tdTomato, magenta). Nuclei are shown in white (SPY620). Time in minutes. Scale bar, 50 µm. Related to Fig. 9i.

**Supplementary Video 15: Fibrotic-like assembloids show disruption of cell integrity.**

Maximum intensity projection of a 3D assembloid showing big bursts of cell-free DNA signal, imaging from 2 days after seeding. Time in hours. Nuclei are depicted in white (stained with SPY620), mesenchyme cells in green (nuc-GFP, Pdgfra-H2BGF) and cholangiocytes in magenta (mem-tdTomato). Brightfield is also shown. Scale bar, 100 µm.

**Supplementary Video 16: Fibrotic-like assembloids show no functional bile duct-bile canaliculi connection.**

Live imaging of the bile acid analogue cholyl-L-lysyl-fluorescein (CLF, pseudo-colour range) in fibrotic-like assembloids is not observed. Cholangiocytes (nuc-tdTomato, magenta) and mesenchyme (nuc-GFP, green), and brightfield are also shown. Time in minutes. Scale bar, 50 µm.
