# Supplementary material for: Mouse liver assembloids model periportal architecture and biliary fibrosis
Source: Nature. 2025 May 29;644(8076):473–82. doi: 10.1038/s41586-025-09183-9 (PMC12350178; doi:10.1038/s41586-025-09183-9)
Supplement: Supplementary file 2 — Reporting Summary [file 41586_2025_9183_MOESM2_ESM.pdf]

nature portfolio

Corresponding author(s): Meritxell Huch

Last updated by author(s): May 7, 2025

Reporting Summary

Nature Portfolio wishes to improve the reproducibility of the work that we publish. This form provides structure for consistency and transparency in reporting. For further information on Nature Portfolio policies, see our Editorial Policies and the Editorial Policy Checklist.

Statistics

For all statistical analyses, confirm that the following items are present in the figure legend, table legend, main text, or Methods section.

n/a	Confirmed	
☐	☒	The exact sample size (*n*) for each experimental group/condition, given as a discrete number and unit of measurement
☒	☐	A statement on whether measurements were taken from distinct samples or whether the same sample was measured repeatedly
☐	☒	The statistical test(s) used AND whether they are one- or two-sided<br>*Only common tests should be described solely by name; describe more complex techniques in the Methods section.*
☒	☐	A description of all covariates tested
☐	☒	A description of any assumptions or corrections, such as tests of normality and adjustment for multiple comparisons
☐	☒	A full description of the statistical parameters including central tendency (e.g. means) or other basic estimates (e.g. regression coefficient) AND variation (e.g. standard deviation) or associated estimates of uncertainty (e.g. confidence intervals)
☐	☒	For null hypothesis testing, the test statistic (e.g. *F*, *t*, *r*) with confidence intervals, effect sizes, degrees of freedom and *P* value noted<br>*Give P values as exact values whenever suitable.*
☒	☐	For Bayesian analysis, information on the choice of priors and Markov chain Monte Carlo settings
☒	☐	For hierarchical and complex designs, identification of the appropriate level for tests and full reporting of outcomes
☐	☒	Estimates of effect sizes (e.g. Cohen's *d*, Pearson's *r*), indicating how they were calculated

Our web collection on statistics for biologists contains articles on many of the points above.

Software and code

Policy information about availability of computer code

Data collection	The following software was used for data collection: scRNAseq data - NovaSeq S4 v1.5 4XP (200cyc); Confocal Imaging: Zeiss ZEN 3.6 celldiscoverer (version 3.6.095.03000); Zen 2011 SP7 FP3 (black), version 14.0.25.201 (780 Zeiss inverted microscope); Zen Black 2011 SP7 FP3 64bit; version 14.0.27.201 (Zeiss 780 upright microscope); ZEN 2.3 SP1 FP3 (black) v14.0.25.201 (Zeiss 880 inverted microscope); ZEN 2.3 SP1 FP3 (black) v14.0.27.201 (Zeiss 880 upright microscope); Timelapse imaging - Cell Voyager Measurement System Release R1.17.05 (Yokogawa CV7000 MPO); Lightsheet imaging - Viventis Microscope and Environment v.2.0.0.2 ; Brightfield imaging - Leica Application Suite v.4.6.0 (Leica DMIL LED microscope) or v.4.13.0 (Leica M80 microscope) ; qPCR - LightCycler 96 software (Version: 1.1.0.1320) or QuantStudio7 software (QS 6/7 Pro v1.8.1); cytokine array - iBright Analysis Software v.5.2.0; Albumin ELISA and bile acid assay - Perkin Elmer Envision 2104 EnVision Manager v.1.13.3009.1401, Flow cytometry - BD FACSDiva v.8.0.2, Sony Cell Sorter Software v.3.1.2.
Data analysis	Confocal and light sheet imaging data was analysed with FIJI v.2.14.0/1.54f, and segmented using Arivis Vision 4D (Version: 4.1.0. Build: 16702. 20200324) or Motion Tracking v. 8.100.6 (http://motiontracking.mpi-cbg.de). Data analysis of bile canaliculi was performed using a custom script in FIJI (https://git.mpi-cbg.de/huch_lab/assembloid-paper) . For Yokogawa timelapse imaging, data was assembled using two FIJI macros "Macro CV7000 Make Field Montages" and "Macro CV7000 Make Timelapse Movies", which are publicly available (https://github.com/stoeter/Fiji-Tools-for-HCS). Data presentation and statistical analysis performed with GraphPad Prism v10 (v10.0.2 and v.10.0.0) for non-sequencing data. For Flow cytometry data, Flowjo v.10.8.1 was used to analyse and visualise the data.<br><br>Code used for data analysis and visualisation of scRNAseq dataset included: Cellbender (0.3.0), bcl2fastq2 (2.20.0), CellRanger v.7.1.0, scanpy v.1.9.2, GSEApy v.1.0.5, plotnine v.0.12.3, R v.4.2.0, ggraph v.2.1.0 and LIANA v.0.1.9, Scrublet (https://github.com/swolock/scrublet). For bulk RNAseq analysis, featureCount (v2.0.6) was used to assigned reads exons, transcripts and CDS. Differential gene expression analysis was performed using the R (4.2.0) package DESeq2 (1.36.0). Gene set enrichment analysis for bulk RNAseq data was performed using the R

package fgsea (1.22.0).

For manuscripts utilizing custom algorithms or software that are central to the research but not yet described in published literature, software must be made available to editors and reviewers. We strongly encourage code deposition in a community repository (e.g. GitHub). See the Nature Portfolio guidelines for submitting code & software for further information.

Data

Policy information about availability of data

All manuscripts must include a data availability statement. This statement should provide the following information, where applicable:

- Accession codes, unique identifiers, or web links for publicly available datasets
- A description of any restrictions on data availability
- For clinical datasets or third party data, please ensure that the statement adheres to our policy

The scRNAseq and bulk RNAseq datasets generated during this study are available at Gene Expression Omnibus (GEO, https://www.ncbi.nlm.nih.gov/geo/info/seq.html) under the accession number GSE274971 and GSE274973 . The full lists of bulk RNaseq TPMs and DEG are found in Supplementary Table 2 and Supplementary Table 6, respectively. All GSEA terms are available in Supplementary Table 3 and Supplementary Table 5. All inferred cell-cell interactions are available in Supplementary Supplementary Table 4.

The scRNAseq data was aligned to the GRCm39 (release 109) mouse genome with the 10x's cellranger's (7.1.0) count. Bulk RNAseq data was aligned to the mouse genome GRCm39 release 109 using STAR aligner (2.7.11b). All other images, qPCR and measurement data are presented within the manuscript, and data used to plot graphs is provided as Source Data files for each figure.

For Gene Set Enrichment Analysis (GSEA), the following databeses were used: the Whole organoid GSEA between healthy and fibrotic conditions was performed for the MSigDB hallmark (2020), WikiPathways (2019, Mouse), Reactome (2022), Elsevier Pathway Collection and GO Molecular Function (2023) gene sets; Cell type specific GSEA between conditions was performed for the MSigDB hallmark (2020) gene set. Functional identity was assessed by GSEA by comparing each cell type to all other cell types using the GO Molecular Function (2023), GO Biological Process (2023) and GO Cellular Component (2023) gene sets.

Research involving human participants, their data, or biological material

Policy information about studies with human participants or human data. See also policy information about sex, gender (identity/presentation), and sexual orientation and race, ethnicity and racism.

Reporting on sex and gender	Not applicable (N/A) - no human participants, data or biological material was used for this study.
Reporting on race, ethnicity, or other socially relevant groupings	N/A
Population characteristics	N/A
Recruitment	N/A
Ethics oversight	N/A

Note that full information on the approval of the study protocol must also be provided in the manuscript.

Field-specific reporting

Please select the one below that is the best fit for your research. If you are not sure, read the appropriate sections before making your selection.

☒ Life sciences ☐ Behavioural & social sciences ☐ Ecological, evolutionary & environmental sciences

For a reference copy of the document with all sections, see nature.com/documents/nr-reporting-summary-flat.pdf

Life sciences study design

All studies must disclose on these points even when the disclosure is negative.

Sample size	No statistical methods were used to calculate sample size and group size. For cell culture experiments we follow the recommendations from ISSCR guidelines published in Ludwig et al. 2023 (PMID: 37703820).
Data exclusions	scRNAseq thresholding involved excluding cells with abnormally high or low transcripts, and based on high mitochondrial gene content as is standard practice for downstream analysis of this sequencing data. We also excluded doublets to improve stringency, as described in the methods.
Replication	All experiments were replicated as indicated in the figure legend.
Randomization	Experimental procedures always involved processing control and experimental samples in a random order, rather than by condition.

Blinding	We did not perform blinding for experiments, as blinding was considered not to affect the measurement result. Whenever possible, investigators were blinded performing the experimental analysis.

Reporting for specific materials, systems and methods

We require information from authors about some types of materials, experimental systems and methods used in many studies. Here, indicate whether each material, system or method listed is relevant to your study. If you are not sure if a list item applies to your research, read the appropriate section before selecting a response.

Materials & experimental systems

n/a	Involved in the study
☐	☒ Antibodies
☐	☒ Eukaryotic cell lines
☒	☐ Palaeontology and archaeology
☐	☒ Animals and other organisms
☒	☐ Clinical data
☒	☐ Dual use research of concern
☒	☐ Plants

Methods

n/a	Involved in the study
☒	☐ ChIP-seq
☐	☒ Flow cytometry
☒	☐ MRI-based neuroimaging

Antibodies

Antibodies used

List of antibodies and dyes used in this study.
Primary antibodies:
Rabbit monoclonal anti-E-cadherin (24E10), Cell Signaling # 3195 RRID:AB_2291471
Rat monoclonal anti-E-cadherin (ECCD-2), Thermo Fisher Scientific # 131900 RRID:AB_2533005
Rat monoclonal anti-CD13 (ER-BMDM1), Novus # NB100-64843 RRID:AB_959651
Rat monoclonal anti-Cytokeratin-19 (TROMA-III), Sigma-Aldrich # MABT913 RRID:AB_2892523
Rabbit polyclonal anti-ZO-1, Thermo Fisher Scientific # 40-2200 RRID:AB_2533456
Goat polyclonal anti-Albumin, Novus # NB600-41532 RRID:AB_805588
Rabbit monoclonal anti-Radixin (C4G7), Cell Signaling # 2636 RRID:AB_2238294
Rabbit polyclonal anti-Cytokeratin, Dako # Z0622 RRID:AB_2650434
Rabbit monoclonal anti-Hnf4-alfa (EPR16885), Abcam # ab181604 RRID:AB_2890918
Rabbit monoclonal anti-SOX9 (EPR14335-78), Abcam # ab185966 RRID:AB_2728660
Goat anti-Osteopontin Polyclonal, R&D Systems # AF808 RRID: AB_2194992
Rat anti-CD34 Monoclonal Antibody (RAM34), eBioscience™ ThermoFisher Scientific # 14-0341-85 RRID:AB_467211
Rabbit anti-Cleaved Caspase-3 (Asp175) monoclonal (Clone 5A1E), Cell Signaling Technology # 9664 RRID: AB_2070042
Rat Purified anti-mouse Ly-6A/E (Sca-1), BioLegend # 108102 RRID:AB_313339
Rabbit Polyclonal anti-Transcription Factor Sox-9, Millipore # AB5535 RRID:AB_2239761
Mouse monoclonal anti-Vimentin (clone LN-6), Merck # V2258 RRID:AB_261856
Rat anti-Ly-6A/E (Sca-1) monoclonal (Clone D7), Super Bright 436, ThermoFisher Scientific # 62-5981-82 RRID: AB_2637287
Rat anti- CD326 (EpCAM) monoclonal (Clone G8.8), APC, ThermoFisher Scientific # 17-5791-80 RRID: AB_2734965
Rat anti-CD11b monoclonal (Clone M1/70), PE-Cy7, BD Biosciences # 552850 RRID: AB_394491
Rat anti-CD31 monoclonal (Clone 390), PE-Cy7, BD Biosciences # 561410 RRID: AB_10612003
Rat anti-CD45 monoclonal (Clone 30-F11), PE-Cy7, BD Biosciences # 552848 RRID: AB_394489
Rabbit anti-Elastin (polyclonal IgG), Cedarlane Laboratories Limited #CL55041AP RRID: AB_10061195
Rabbit polyclonal anti-Glutamine Synthetase (GS), Sigma-Aldrich # G2781 RRID: AB_259853
Rabbit polyclonal anti-Cytochrome P450 2E1 (CYP2E1), Abcam # ab28146 RRID: AB_2089985
Rabbit anti-Ki67 monoclonal (Clone SP6), ThermoFisher Scientific #RM 9106 S1 RRID:AB_2341197

Secondary antibodies
Donkey anti-Rat IgG (H+L) Highly Cross-Adsorbed Secondary Antibody, Alexa Fluor 488, ThermoFisher Scientific # A-21208 RRID:AB_2535794
Donkey anti-Rat IgG (H+L) Highly Cross-Adsorbed Secondary Antibody, Alexa Fluor Plus 555, ThermoFisher Scientific # A48270 RRID:AB_2896336
Donkey anti-Rat IgG (H+L) Highly Cross-Adsorbed Secondary Antibody, Alexa Fluor 594, ThermoFisher Scientific # A-21209 RRID:AB_2535795
Goat anti-Rat IgG (H+L) Cross-Adsorbed Secondary Antibody, Alexa Fluor 647, ThermoFisher Scientific # A-21247 RRID:AB_141778
Goat anti-Rabbit IgG (H+L) Cross-Adsorbed Secondary Antibody, Alexa Fluor 532, ThermoFisher Scientific # A-11009 RRID:AB_2534076
Donkey anti-Rabbit IgG (H+L), highly cross-adsorbed, CF™ 633 antibody, Merck # SAB4600132
Donkey anti-Rabbit IgG (H+L) Highly Cross-Adsorbed Secondary Antibody, Alexa Fluor 647, ThermoFisher Scientific # A-31573 RRID:AB_2536183
Donkey anti-Mouse IgG (H+L) Polyclonal Antibody, Alexa Fluor 647, ThermoFisher Scientific # A-31571 RRID:AB_162542
Donkey anti-Goat IgG (H+L) Cross-Adsorbed Secondary Antibody, Alexa Fluor 594, ThermoFisher Scientific # A-11058 RRID:AB_2534105
Donkey anti-Goat IgG (H+L) Cross-Adsorbed Secondary Antibody, Alexa Fluor 647, ThermoFisher Scientific # A-21447 RRID:AB_2535864
Donkey Anti-Rat IgG (H+L), Highly Cross-Adsorbed, CF 568, Biotium # 20092 RRID:AB_10855000

Dyes
SiR-Actin, Spirochrome # SC001
Corning® Cholyl-Lysyl-Fluorescein (CLF), Corning # 451041
SPY620-DNA, Spirochrome # SC401
DAPI, BD Biosciences # BD564907
Phalloidin 647, ThermoFisher Scientific # A22287
Phalloidin 488, ThermoFisher Scientific # A12379
CellTracker™ Green CMFDA Dye (Chloromethylfluorescein diacetate), ThermoFisher Scientific #C2925
16:0-06:0 NBD PC fluorescent lipid (phosphatidylcholine), Avanti Polar lipids, Merck #810130P-1mg
5-CFDA, AM (5-Carboxyfluorescein Diacetate, Acetoxymethyl Ester), ThermoFisher Scientific #C1354
SPY555 DNA, Spirochrome #SC201

Blocking antibodies
Normal Goat IgG Control R&D Systems, Biotechne #AB-108-C RRID:AB_354267
Normal Rat IgG Control (Azide Free), R&D Systems, Biotechne #6-001-F RRID:AB_2616570
Mouse IgG1 Isotype Control, R&D Systems, Biotechne #MAB002 RRID:AB_357344
Mouse uPAR Antibody R&D Systems, Biotechne #AF534-SP RRID:AB_2165351
Human/Mouse TNF-alpha Antibody, R&D Systems, Biotechne #AF410-SP RRID:AB_354479
Mouse CX3CL1/Fractalkine Chemokine Domain Antibody, R&D Systems, Biotechne #AF472-SP RRID:AB_2276839
Mouse CCL11/Eotaxin Antibody, R&D Systems, Biotechne #AF420-SP RRID:AB_354486
Human/Mouse CXCL12/SDF-1 Antibody, R&D Systems, Biotechne #MAB310-SP RRID:AB_2276927
BD Pharmingen™ Purified Rat Anti-Mouse CD47 Clone miap301 unlabelled Antibody, BD Biosciences #555297 RRID:AB_395713
Human/Mouse CXCL12/SDF-1 Antibody, R&D Systems, Biotechne #MAB310-SP RRID:AB_2276927
Mouse TIMP-1 Antibody, R&D Systems, Biotechne #AF980-SP RRID:AB_355759
Human/Mouse TIMP-2 Antibody, R&D Systems, Biotechne #AF971-SP RRID:AB_355752
Mouse Osteopontin/OPN Antibody, R&D Systems, Biotechne #AF808-SP RRID:AB_2194992
Mouse CXCL1/GRO alpha /KC/CINC-1 Antibody, R&D Systems, Biotechne #AF-453-SP RRID:AB_354495

Validation

All antibodies are commercially available, and were validated for specificity and application by manufacturers listed here and specified below, or published for the application and species before. Additional information on validation can be found on the manufacturer's websites. Antibodies were used at concentrations suggested in previous published methodologies (https://star-protocols.cell.com/protocols/2730, https://www.cell.com/cell-stem-cell/fulltext/S1934-5909(21)00287-3) or titrated in-house, with the concentration specified in Supplementary Information.

Primary antibodies
anti-E-cadherin (24E10), Cell Signaling # 3195 - WB, IHC, IF, Flow Cytometry; Species reactivity - Mouse, Human;
anti-E-cadherin (ECCD-2),Thermo Fisher Scientific # 131900 - WB, IHC; Species reactivity - Mouse;
anti-CD13 (ER-BMDM1), Novus # NB100-64843 - Flow cytometry, IHC; Species reactivity - Mouse;
anti-Cytokeratin-19 (TROMA-III), Sigma-Aldrich # MABT913 - EM, IF, IHC, IP, WB; Species reactivity - Mouse, Human;
anti-ZO-1, Thermo Fisher Scientific # 40-2200 - WB, IHC, IF; Species reactivity - Mouse, Human, Dog, Rat;
anti-Albumin, Novus # NB600-41532 R- WB, ELISA, ICC/F, IHC; Species reactivity - Mouse;
anti-Radixin (C4G7) ,Cell Signaling # 2636 - WB; Species reactivity - Mouse, Human, Rat, Monkey;
anti-Cytokeratin, Dako # Z0622 - information from manufacturer's website not available, product discontinued;
anti-Hnf4-alfa (EPR16885), Abcam # ab181604 - IP, ChIP, WB, IHC, ChIC; Species reactivity - Mouse, Human, Rat;
anti-SOX9 (EPR14335-78), Abcam # ab185966 - WB, IHC, IF, Flow cytometry; Species reactivity - Mouse, Human, Rat;
anti-Osteopontin, R&D Systems # AF808 - ELISA, WB, IHC, IF, Neutralisation; Species reactivity - Mouse;
anti-CD34 (RAM34), eBioscience™ ThermoFisher Scientific # 14-0341-85 - IHC, Flow cytometry; Species reactivity - Mouse;
anti-Cleaved Caspase-3 (Asp175)(Clone 5A1E), Cell Signaling # 9664 - WB, IP, IHC, IF, Flow cytometry; Species reactivity - Mouse, Human, Rat, Monkey;
anti-Ly-6A/E (Sca-1), BioLegend # 108102 - Flow cytometry, WB, IP, IF, IHC; Species reactivity - Mouse;
anti-Transcription Factor Sox-9, Millipore # AB5535 -WB, IHC; Species reactivity - Mouse, Human, Rat, Chicken;
anti-Vimentin (clone LN-6), Merck # V2258 - IHC, IP, IF, WB; Species reactivity - Mouse, Human, Pig, Sheep, Bovine, Rabbit, Feline, Rat;
anti-Ly-6A/E (Sca-1) (Clone D7), Super Bright 436, ThermoFisher Scientific # 62-5981-82 - Flow cytometry; Species reactivity - Mouse;
anti- CD326 (EpCAM) (Clone G8.8), APC, ThermoFisher Scientific # 17-5791-80 - Flow cytometry; Species reactivity - Mouse;
anti-CD11b (Clone M1/70), PE-Cy7, BD Biosciences # 552850 - Flow cytometry; Species reactivity - Mouse, Human;
anti-CD31 (Clone 390), PE-Cy7, BD Biosciences # 561410 - Flow cytometry; Species reactivity - Mouse;
anti-CD45 (Clone 30-F11), PE-Cy7, BD Biosciences # 552848 - Flow cytometry; Species reactivity - Mouse;
anti-Elastin (polyclonal IgG), Cedarlane Laboratories Limited #CL55041AP - information from manufacturer's website not available, product discontinued;
anti-Glutamine Synthetase (GS), Sigma-Aldrich # G2781 - WB, IHC; Species reactivity - Rat;
anti-Cytochrome P450 2E1 (CYP2E1), Abcam # ab28146 - WB, IF; Species reactivity - Mouse, Human, Rat, Rabbit;
anti-Ki67 (Clone SP6), ThermoFisher Scientific #RM 9106 S1 - IHC; Species reactivity - Human;

Secondary antibodies
Donkey anti-Rat IgG (H+L) AF-488, ThermoFisher Scientific # A-21208 - IHC, IF; Species reactivity - Rat;
Donkey anti-Rat IgG (H+L) AF-Plus-555, ThermoFisher Scientific # A48270 - IF; Species reactivity - Rat;
Donkey anti-Rat IgG (H+L) AF-594, ThermoFisher Scientific # A-21209 - IHC, IF; Species reactivity - Rat;
Goat anti-Rat IgG (H+L) AF-647, ThermoFisher Scientific # A-21247 - WB, IP, IHC, IF; Species reactivity - Rat;
Goat anti-Rabbit IgG (H+L) AF-532, ThermoFisher Scientific # A-11009 - WB, IF; Species reactivity - Rabbit;
Donkey anti-Rabbit IgG (H+L), CF™ 633, Merck # SAB4600132 - Flow cytometry, IHC, IF; Species reactivity - Rabbit;
Donkey anti-Rabbit IgG (H+L) AF-647, ThermoFisher Scientific # A-31573 - WB, IHC, IF; Species reactivity - Rabbit;
Donkey anti-Mouse IgG (H+L) AF-647, ThermoFisher Scientific # A-31571 - Flow cytometry, IF; Species reactivity - Mouse;

Donkey anti-Goat IgG (H+L) AF-594, ThermoFisher Scientific # A-11058 - Flow cytometry, IHC, IF; Species reactivity - Goat;
Donkey anti-Goat IgG (H+L) AF-647, ThermoFisher Scientific # A-21447 - IHC, IF; Species reactivity - Goat;
Donkey anti-Rat IgG (H+L) CF 568, Biotium # 20092 - Flow cytometry, IHC, IF, WB; Species reactivity - Rat;

Blocking antibodies
Goat IgG Control R&D Systems, Biotechne #AB-108-C - Flow cytometry, IF; Species reactivity - Goat;
Rat IgG Control (Azide Free) R&D Systems, Biotechne #6-001-F - Control applications - no other validation specified; Species reactivity - Rat;
Mouse IgG1 Control R&D Systems, Biotechne #MAB002 - Flow cytometry; Species reactivity - Mouse;
Mouse uPAR Antibody R&D Systems, Biotechne #AF534-SP - Blocade of Receptor-Ligand Interaction, CyTOF, Flow cytometry, WB, IHC; Species reactivity - Mouse;
Human/Mouse TNF-alpha Antibody R&D Systems, Biotechne #AF410-SP - CyTOF, ELISA capture, IHC,IF, Flow cytometry, Neutralisation, WB; Species reactivity - Mouse, Human;
Mouse CX3CL1/Fractalkine Chemokine Domain Antibody, R&D Systems, Biotechne #AF472-SP - Flow cytometry, IHC, IF, Neutralisation, WB; Species reactivity - Mouse;
Mouse CCL11/Eotaxin Antibody, R&D Systems, Biotechne #AF420-SP - ELISA capture, IHC, Neutralisation, WB; Species reactivity - Mouse;
Human/Mouse CXCL12/SDF-1 Antibody, R&D Systems, Biotechne #MAB310-SP - Neutralisation; Species reactivity - Mouse, Human;
Rat Anti-Mouse CD47 (miap301) Antibody, BD Biosciences #555297 - Flow cytometry, IF, IHC; Species reactivity - Mouse;
Mouse TIMP-1 Antibody, R&D Systems, Biotechne #AF980-SP - IHC, IP, Neutralisation, WB; Species reactivity - Mouse;
Human/Mouse TIMP-2 Antibody, R&D Systems, Biotechne #AF971-SP - ELISA, WB; Species reactivity - Mouse, Human;
Mouse Osteopontin/OPN Antibody, R&D Systems, Biotechne #AF808-SP - ELISA Capture, IHC, IF, Neutralisation, WB; Species reactivity - Mouse;
Mouse CXCL1/GRO alpha /KC/CINC-1 Antibody R&D Systems, Biotechne #AF-453-SP - Neutralisation, WB; Species reactivity - Mouse.

Eukaryotic cell lines

Policy information about cell lines and Sex and Gender in Research

Cell line source(s)	The mesenchymal cells and organoid lines are all primary material derived from isolated livers by investigators in this study.
Authentication	N/A
Mycoplasma contamination	Mycoplasma contamination was regularly tested on all cell and organoid laboratory lines throughout this study, using MycoAlert® Mycoplasma Detection Kit (Lonza #LT07-118).
Commonly misidentified lines (See ICLAC register)	No ICLAC lines were used in this study.

Animals and other research organisms

Policy information about studies involving animals; ARRIVE guidelines recommended for reporting animal research, and Sex and Gender in Research

Laboratory animals	Mouse experiments were performed in accordance with the German animal welfare legislation and in strict pathogen-free conditions in the animal facility of the MPI-CBG. Protocols were approved by the Institutional Animal Welfare Officer (Tierschutzbeauftragter), and all necessary licenses were obtained from the regional Ethical Commission for Animal Experimentation of Dresden, Germany (Tierversuchskommission, Landesdirektion Dresden). The MPI-CBG's laboratory animal housing is exclusively barrier housing. All mice are kept in IVC systems (individually ventilated cages) under a 12:12-hour light/dark cycle. The animal room temperature is between 20 and 24 C and the relative humidity is 55±10%. Both are subject to constant monitoring. Sterile food and water were given ad libitum. Healthy adult mice (8-25 weeks of age) of both sexes were used for experiments. For MDR2 experiments, 8 weeks of age were used. WT, C57/Bl6 mice, Rosa26-mTmG, Rosa26-nTnG, PDGFRα-H2B-GFP, PDGFRα-H2B-GFP x Rosa26-mTmG, Prom1-CreERT2 x R26-LSL-ZsGreen, Itgb1-fl/fl x R26-LSL-ZsGreen or MDR2-KO mice were used for experiments. Rosa26-mTmG [Gt(ROSA)26Sortm4(ACTB-tdTomato,-EGFP)Luo/J] and Rosa26-nTnG [B6;129S6-Gt(ROSA)26Sortm1(CAG-tdTomato*,-EGFP*)Ees/J] were obtained from the Jackson Laboratory (JAX). The PDGFRα-H2B-GFP ([B6.129S4-Pdgfratm11(EGFP)Sor/J] was described previously and obtained from Prof. Magdalena Zernicka-Goetz. The PDGFRα-H2B-GFP x Rosa26-mTmG was generated by crossing the PDGFRα-H2B-GFP with the Rosa26-mTmG obtained and described above. The MDR2-knock out line [FVB.129P2-Abcb4tm1Bor/J] was described before. The R26-LSL-ZsGreen B6.Cg-Gt(ROSA)26Sortm6(CAG-ZsGreen1)Hze/J) was obtained from JAX. Itgb1-fl/fl (B6;129-Itgb1tm1Efu/J) was described previously, and obtained from Prof. Magdalena Zernicka-Goetz. Itgb1-fl/fl x R26-ZsGreen was generated by crossing the Itgb1-fl/fl with the R26-LSL-ZsGreen. The Prom1-CreERT2 x R26-LSL-ZsGreen was generated by crossing the Prom1-CreERT2 (B6N;129S-Prom1tm1(cre/ERT2)Gilb/J) -described previously and obtained from Prof Richard Gilbertson- with the R26-LSL-ZsGreen. The Confetti mouse (Gt(ROSA)26Sortm1(CAG-Brainbow2.1)Cle/J) was described before and recombined in vitro to obtain mCFP cholangiocyte organoids. Mice were bred onto a C57/B6 background.
Wild animals	This study did not involve wild animals.
Reporting on sex	Both male and female mice were used in this study indistinctively.
Field-collected samples	This study did not involve field-collected samples.

Ethics oversight	Mouse experiments were performed in accordance with the German animal welfare legislation and in strict pathogen-free conditions in the animal facility of the MPI-CBG. Protocols were approved by the Institutional Animal Welfare Officer (Tierschutzbeauftragter), and all necessary licenses were obtained from the regional Ethical Commission for Animal Experimentation of Dresden, Germany (Tierversuchskommission, Landesdirektion Dresden).

Note that full information on the approval of the study protocol must also be provided in the manuscript.

Flow Cytometry

Plots

Confirm that:

☒ The axis labels state the marker and fluorochrome used (e.g. CD4-FITC).

☒ The axis scales are clearly visible. Include numbers along axes only for bottom left plot of group (a 'group' is an analysis of identical markers).

☒ All plots are contour plots with outliers or pseudocolor plots.

☒ A numerical value for number of cells or percentage (with statistics) is provided.

Methodology

Sample preparation	For the analysis of cholangiocytes from hepatocyte isolation and HepOrg culture, primary hepatocytes and other liver cells were isolated from mice as described in Methods Section. The cells were then strained with 100 µM strainer, washed one time with AdDMEM/F12 (ThermoFisher, 12634010) medium containing 1% HEPES (ThermoFisher, #15630-056), 1% Penicillin/Streptomycin (ThermoFisher, #15140-122), Glutamax (ThermoFisher, #35050-068), spun 5min at 100g and stained 30min with following antibody against EpCAM, conjugated to APC (CD326 (EpCAM) Monoclonal Antibody (G8.8), APC, eBioscience, ThermoFisher, #17-5791-80). Before sorting, cells were washed one time with above medium, spun 5min at 100g and resuspended in the same medium for sorting. In case of cholangiocytes from HepOrg culture, all cells from the culture were collected in above media, spun 5min at 200g and dissociated by incubation with TrypLE Express (ThermoFisher, #12605010) for 5 min at 37°C, before being strained and stained as outlined above.
Instrument	Sony MA900 Multi-Application Cell Sorter, FacsAria Fusion Cell Sorter
Software	BD FACSDiva v.8.0.2, Sony Cell Sorter Software v.3.1.2, Flowjo v.10.8.1
Cell population abundance	The final sorted population was above 90% of the total detected events. Single cells were 75-81% of the final sorted population. EpCAM+ population was 0.08% of total events in the hepatocyte isolation prep. EpCAM+ population was 19-47% of all single cells in the hepatocyte organoid culture prep. The proportions of ZsGreen negative and positive fractions are specified in Extended Data Figure 4e. The mode of 4-way purity was always selected for cell sorts.
Gating strategy	The starting population was determined based on the FSC-A and SSC-A statuses, and single cells were gated based on FSC-H and FSC-W intensities. All single cells were gated on positive staining for the EpCAM-APC antibody, as determined by the negative unstained control. Then, EpCAM-positive population was gated on ZsGreen positive or negative fluorescence, as determined by negative control with ZsGreen expression.

☒ Tick this box to confirm that a figure exemplifying the gating strategy is provided in the Supplementary Information.
