# Supplementary figures and images for: Mouse liver assembloids model periportal architecture and biliary fibrosis
Source: Nature. 2025 May 29;644(8076):473–82. doi: 10.1038/s41586-025-09183-9 (PMC12350178; doi:10.1038/s41586-025-09183-9)

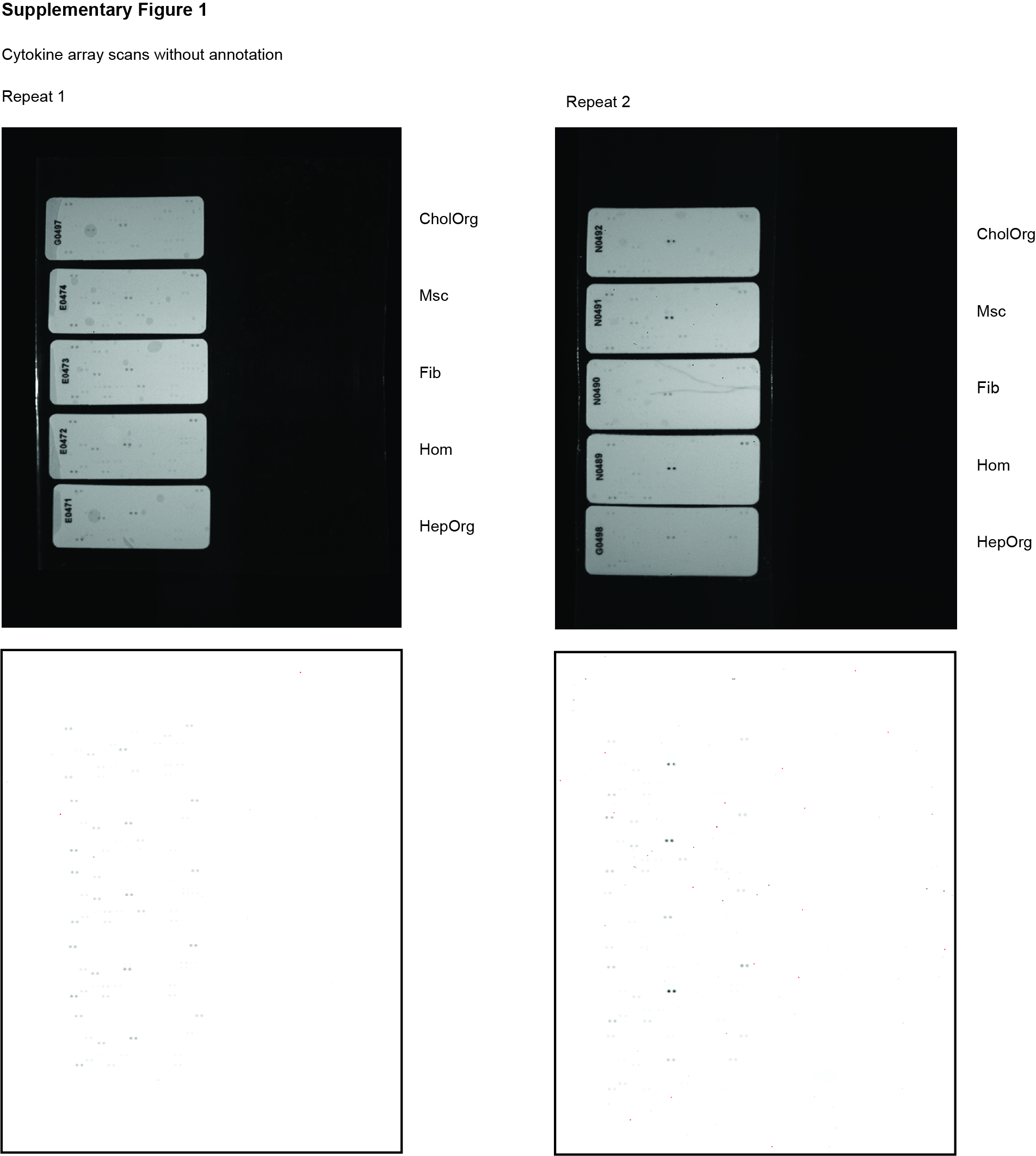

Supplement: Supplementary file 3 — Cytokine array raw data. Cytokine array blots from 2 biological replicates, presented as raw scans of the signal, and overlay with the blot brightfield image. Images are annotated for the sample name. Supernatants from monocultures of CholOrg, or HepOrg or mesenchyme cells or from assembloids from homeostatic or fibrotic-like conditions were analysed. [file 41586_2025_9183_MOESM3_ESM.jpg]
